# Determinants of Antimicrobial Resistance among the Different European Countries: More than Human and Animal Antimicrobial Consumption

**DOI:** 10.3390/antibiotics10070834

**Published:** 2021-07-09

**Authors:** Ana C. Silva, Paulo Jorge Nogueira, José-Artur Paiva

**Affiliations:** 1 Institute of Environmental Health (ISAMB), Faculdade de Medicina, Universidade de Lisboa, Avenida Professor Egas Moniz, 1049-028 Lisboa, Portugal; pnogueira@medicina.ulisboa.pt; 2 National Authority of Medicines and Health Products, I.P. (Infarmed, I.P.), Av Brasil 53, 1749-004 Lisboa, Portugal; 3 Laboratory of Biomathmatics, Institute of Preventive Medicine and Public Health (IMPSP), Faculdade de Medicina, Universidade de Lisboa, Avenida Professor Egas Moniz, 1049-028 Lisboa, Portugal; 4 Department of Intensive Care Medicine, Centro Hospitalar Universitário São João, Alameda Professor Hernâni Monteiro, 4200-319 Porto, Portugal; jarturpaiva@gmail.com; 5 Department of Medicine, Faculty of Medicine, University of Porto, Alameda Professor Hernâni Monteiro, 4200-319 Porto, Portugal; 6 Grupo de Infeção e Sépsis (GIS), Alameda Professor Hernâni Monteiro, 4000-000 Porto, Portugal; 7 Programa de Prevenção e Controlo de Infeções e de Resistência aos Antimicrobianos (PPCIRA), Directorate General of Health, Alameda D. Afonso Henriques 45, 1049-005 Lisboa, Portugal

**Keywords:** antimicrobial resistance, One Health, antibiotic consumption, healthcare expenditure, health system, socio-economic determinants

## Abstract

Although antimicrobial consumption is considered the main driver of antimicrobial resistance (AMR), other factors probably have a significant but less studied impact. The study’s goal was to assess AMR drivers across different European countries and quantify their possible contributions using the latest data available. Using the ESAC-Net (European Surveillance of Antimicrobial Consumption Network) database, the ESVAC (European Surveillance of Veterinary Antimicrobial Consumption) database and the OECD (Organisation for Economic Cooperation and Development) information, a dataset including 23 European countries was created. Associations between AMR and potential contributing factors were assessed using bivariate correlation and multiple linear regression models for multivariable analyses. Factors associated with the AMR rate among European countries were human ambulatory consumption of antibiotics and per capita expenditure on health, meaning that the higher human ambulatory consumption of antibiotics and the lower the per capita expenditure on health, the higher the AMR. Both variables together explain 74% of AMR variation. Private expenditure on health in terms of % GDP (Gross Domestic Profit) was positively related to a higher AMR rate. In conclusion, considering antibiotic consumption as the most important factor contributing to AMR may be a deviant focus, as resistance transmission may be paramount for AMR levels. Low per capita expenditure on health, probably a surrogate of worse healthcare conditions and a high level of resistance transmission, has a strong correlation with the AMR rate. Increasing public expenditure on healthcare, to strengthen infection control structures and processes interventions, seems relevant to tackle antimicrobial resistance at the European scale.

## 1. Introduction

Antimicrobial resistance (AMR) is a significant cause of morbidity and mortality globally [1,2,3]. Multidrug-resistant microorganisms spread rapidly, and AMR is recognised as a “One Health problem” [4], which raises an enormous threat to society. Each year, AMR is responsible for about 33,000 deaths and costs about 1.1 billion Euros to the health care systems of EU/EEA countries [5]. Between 2007 and 2015, in the EU/EEA, the health burden of infections due to bacteria with AMR has increased. The number of deaths attributable to infections due to *K. pneumoniae* resistant to carbapenems increased six-fold. The number of deaths attributable to infections due to third-generation cephalosporin-resistant *E. coli* increased four-fold [5]. Currently, close to one in five infections in the EU/EEA is due to antibiotic-resistant bacteria. If no effective action is put in place, AMR to second-line antibiotics will be 72% higher in 2030 compared to 2005, in the EU/EEA. In the same period, AMR to last-line treatments will more than double [5].

Antibiotic consumption is considered the main force driving the increase in AMR [5]. The association between antibiotic consumption and resistance is well documented across spatial and temporal scales at individual hospitals, communities and countries [6,7,8]. Between 2000 and 2015, antibiotic consumption, expressed in defined daily doses (DDD), increased 65% (21.1–34.8 billion DDDs), and the antibiotic consumption rate increased 39% (11.3–15.7 DDDs per 1000 inhabitants per day), around the globe [9].

However, other factors may contribute to an increased prevalence of AMR. In fact, although antibiotic usage is an important driver for increasing antimicrobial resistance levels, the spreads of resistant bacteria or the genes that encode resistance are likely to be at least as important in the dissemination and prevalence of antimicrobial resistance [10]. The quality and performance of health systems and several socio-economic factors may significantly impact the rate of transmission of multidrug-resistant microorganisms and even in the etiquette of antibiotic utilization [11]. Study and understand the heterogeneity of socio-economic conditions and health systems across Europe and the huge variation in AMR rate among European countries may allow us to acknowledge better the relative importance of different possible drivers of AMR.

There are a few studies that assess the impact of socioeconomic conditions and health systems organization on AMR, at the same time they assess human and animal consumption, so it makes sense to conduct this study aiming to assess the relative weight of different variables on explaining the heterogeneity of AMR among European countries.

The European countries included in our research were: Austria, Belgium, Czech Republic, Estonia, Finland, France, Germany, Greece, Hungary, Iceland, Ireland, Italy, Latvia, Lithuania, Luxembourg, The Netherlands, Norway, Poland, Portugal, Slovakia, Slovenia, Spain and the United Kingdom.

## 2. Materials and Methods 

Using three sources of data (ESAC-Net database, ESVAC database and OECD Stats) we created a dataset for 23 European countries.

### 2.1. Data

Data for antimicrobial resistance and healthcare-associated infections were obtained from the Health at a Glance 2019 [12] report, based on the ECDC report on point prevalence survey [13]. A composite index of AMR evaluated antimicrobial resistance. The composite index of AMR was calculated as the percentage of resistant isolates for the “first level” AMR markers in the PPS (Point Prevalence Study) protocols, divided by the reported sum of the isolates resulting from antimicrobial susceptibility testing (AST). These first-level markers were: *Staphylococcus aureus* resistant to methicillin (MRSA), *Enterococcus faecium* and *Enterococcus faecalis* resistant to vancomycin, *Enterobacteriaceae* resistant to third-generation cephalosporins, and *Pseudomonas aeruginosa* and *Acinetobacter baumannii* resistant to carbapenems. The percentage of resistant isolates was not calculated when less than 10 isolates, with known AST results, were reported. The composite AMR index at the country level was validated by examining the correlation with the composite AMR index calculated from 2016 EARS-Net data. This includes all index components except for AST results for *Enterobacterales* other than *Escherichia coli* and *Klebsiella pneumonia,* because they are not included in EARS-Net.

Data for human antimicrobial consumption for 2016 was retrieved from the European Surveillance of Antimicrobial Consumption Network (ESAC-Net) database. ESAC-Net (formerly ESAC) is a Europe-wide network of national surveillance systems, providing European reference data on antimicrobial consumption in the human sector. It collects and analyses data on human antimicrobial consumption, both in the community and hospital sector. Data considered refers to antibacterials for systemic use (ATC group J01). (https://www.ecdc.europa.eu/en/antimicrobial-consumption/database/rates-country, accessed on 26 June 2020).

Data for veterinary antimicrobial consumption for 2016 was retrieved from the European Surveillance of Veterinary Antimicrobial Consumption (ESVAC) database. ESVAC is a Europe-wide network of national surveillance systems, providing European reference data on antimicrobial consumption in the veterinary sector. (https://bi.ema.europa.eu/analyticsSOAP/saw.dll?PortalPages, accessed on 26 June 2020).

Data for expenditure on health and years lost for 2016 was retrieved from the Organisation for Economic Cooperation and Development (OECD) website. The OECD is an international organisation that works to build better policies and that collects, analyses and shares data from all its participating countries. Data refers to expenditure on health (all functions), per capita, current prices in Euro (with exchange rate applied at 26 June 2020; Expenditure on health (all functions), share of gross domestic product; Government/compulsory schemes expenditure on health (all functions), share of gross domestic product; and years lost, /100,000 population, aged 75 years old. (https://stats.oecd.org/Index.aspx?DataSetCode=SHA, accessed on 23 June 2020).

### 2.2. Statistical Analysis

A descriptive summary of variables are performed using mean and standard deviations. Correlations among variables are performed using the Pearson R and Spearman Rho correlation coefficient and partial correlations. Multiple linear regression is performed using the stepwise procedure for variable selection to study the variation of the AMR rate across European countries. A two-blocks multiple regression is used to explore the potential non-linear relationships among variables; with the first block fitting a polynomial regression (cubic regression), and the second one using a stepwise adjustment of the remaining variables.

## 3. Results

### 3.1. Univariate Descriptive Statistics

Twenty-three countries were considered, including 21 EU Member States and two EEA countries (Iceland and Norway). With at least one healthcare-associated infection, the average percentage of hospitalized patients was 4.96%, varying between 2.95% and 8.10%. The average proportion of bacteria resistant to antibiotics, isolated from these infections, was 29.9%, with a country range of 10.69% to 68.42%. The statistical results for all the variables included in the study, for the 23 countries, in 2016 are presented in Table 1.

### 3.2. Correlations

Overall bivariate correlation analysis of the several variables with the AMR rate across the European countries reveals that there is a closer, and often significant, correlation with the variables related to expenditure on health than with human or veterinary consumption of antimicrobials (Table 2).

Some non-parametric coefficients (Spearman’s rho) tend to be higher than their respective parametric counterpart (Person’s R). This difference hints at potential non-linear relations of these variables with the AMR rate across European countries.

After adjustment for expenditure on health per capita, human antimicrobials consumption becomes partially highly correlated with the rate of AMR, and this partial correlation is higher for the human ambulatory consumption (*p* = 0.006).

### 3.3. Graphical Visualization

**Figure 1:**
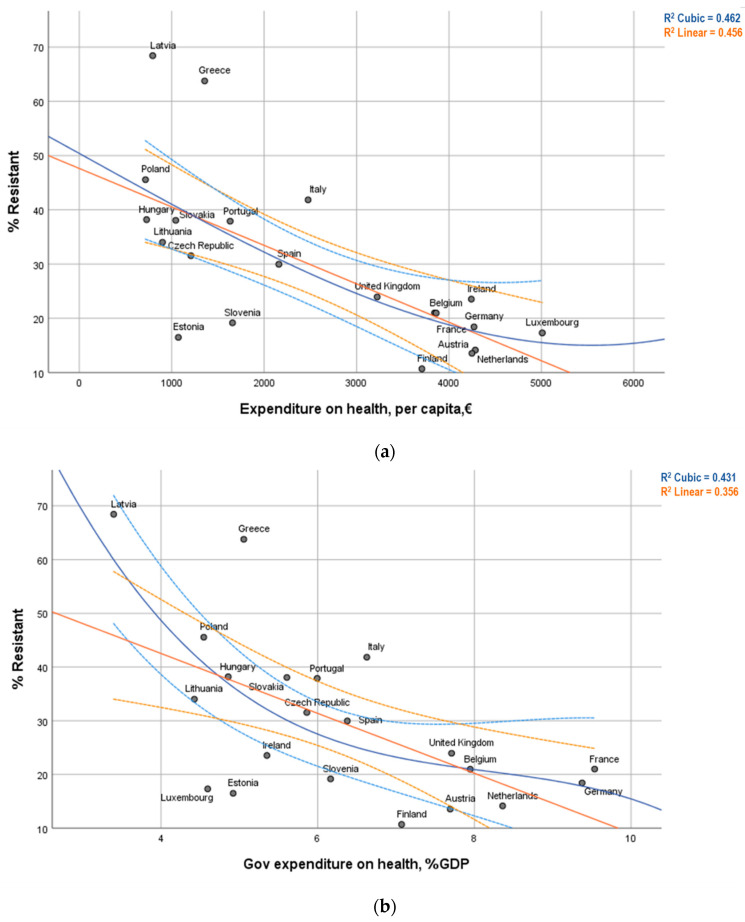
Scatterplot, linear and cubic regression models (and 95% Confidence Intervals) for the relation between Percentual AMR rate with (**a**) Expenditure on health per capita; and (**b**) Government expenditure on health as a percentage of the country GDP.

Figure 1 shows evidence that the AMR rate is inversely related to the country’s per capita expenditure on health, in euros. The relation of a country’s AMR rate with the nation’s per capita expenditure on health shows an almost linear relation (Figure 1a). In contrast, its relation with government expenditure on health, as a percentage of the country’s global GDP tends to be less linear. However, the linear model adjusts rather well to the available data (Figure 1b).

A direct correlation of AMR rate with human ambulatory antimicrobial consumption was also found. Exceptions to this apparent relation are the AMR rate of Latvia (worse than expected) and Finland (better than expected). Graphic visualisation available at Supplementary Materials (Supplementary Materials Figure S1).

A direct relation of AMR rate with years of life lost seems to depart from linearity. However, it does not reach statistical evidence, only a visual hint. Graphic visualisation available at Supplementary Materials (Supplementary Materials Figure S2).

### 3.4. Multiple Linear Regression Model

The results of the multiple linear regression confirm that health expenditure, per capita and human ambulatory antimicrobial consumption, are inversely and directly related to the AMR rate, respectively (Table 3).

### 3.5. Multiple Linear Regression Model

The relation of AMR rate and expenditure on health per capita seemed to be slightly non-linear (Table 2 and Figure 1a), so a two blocks multiple regression analysis were performed. The first block adjusts a cubic model for the AMR rate and expenditure on health per capita relation (Supplementary Materials Table S1). The second block adjusts the remaining variables in the study to explain the remaining variance of the European countries’ AMR rate (Supplementary Materials Table S2). It reveals that private expenditure on health in terms of % GDP is positively related to a higher AMR rate. Human ambulatory consumption of antimicrobials still maintains a positive association with AMR rate, but it noteworthy decreases in importance (smaller standard Beta coefficient).

## 4. Discussion

Our study shows that using a simple stepwise multiple regression analysis, the factors associated with the rate of AMR among European countries are: human ambulatory consumption of antibiotics; and per capita expenditure on health. Meaning that the higher human ambulatory consumption of antibiotics and the lower the per capita expenditure on health are, the higher the AMR is. Both variables together explain 74% of AMR variation. Additionally, the higher the percentage of private health expenditure among global health expenditures is, the higher the rate of antimicrobial resistance is.

This research adds to very few others that emphasise that AMR determinants go far beyond antimicrobial consumption and include socioeconomic and health organisation factors.

The data hints that the relation between the AMR rate and expenditure on health per capita may be non-linear. A more complex regression model was obtained with a potential explanation of 94% of the AMR rate variation across countries. This model, essentially, points to the same relations seen in the simpler model. When accounting for the nonlinearity of the expenditure per capita, the AMR rate reveals an additional monetary investment relation, bringing to the equation the private expenditure on health (positively relating it to AMR) while maintaining some important relation with global ambulatory antimicrobials consumption. This model must carefully be interpreted due to the sample size analysed; nevertheless, it offers itself up to interesting interpretation.

The association between antimicrobial consumption and AMR was demonstrated in several studies, for different pathogens and antimicrobials and in different settings—hospitals, communities, primary care centres, and countries. This kind of context was also reiterated in a recent point prevalence survey in European acute care hospitals, using a composite index of AMR and the percentage of carbapenem-resistant *Enterobacteriaceae* [13]. Conversely, antibiotic stewardship activities, such as reviewing and changing prescriptions when necessary, are negatively associated with AMR. However, this association between antimicrobial consumption and antimicrobial resistance was not found in all studies. Collignon P et al. [14] found that antibiotic consumption was poorly correlated with antimicrobial resistance levels worldwide. However, in developed regions, such as Europe, a correlation between antimicrobial resistance levels and antibiotic usage existed. A possible explanation is that antibiotic volumes are not a factor until a country reaches a medium or high level of success in terms of prevention of transmission of resistant microorganisms or until a certain threshold of antibiotic consumption is reached [15].

The socio-economic development status of a country understandably influences the success of infection control policies. Per capita expenditure on health was also associated with AMR in a recent study carried worldwide [16]. We may speculate that a higher heath expenditure determines better infrastructure, better sanitation, better hygiene, higher nurse-to-patient ratio, better and more frequent professionals’ development, education, a higher level of citizens’ literacy, and less transmission of multidrug-resistant microorganisms. In Collignon P et al. study [14], several infrastructure measures, such as urbanisation, internet accessibility, and access to electricity, were all strongly associated with lower antimicrobial resistance rates, perhaps as surrogates of better sanitation, access to clean water, and access to refrigeration. In that study, similarly to ours, a higher percentage of private healthcare expenditure among global healthcare expenditures was associated with higher levels of antimicrobial resistance. That conclusion was also reached in a previous study comparing only data across Europe [11]. The explanation may come from the fact that the antimicrobials’ prescription is usually less regulated and scrutinised in the private sector. The patients’ craving for the prescription of an antibiotic may be more easily satisfied by doctors. In Brazil, the density of private health clinics was associated with higher antibiotic consumption, which might indicate that private institutions might not restrict the types and quantities of antibiotics as much as in the public sector [16].

One of our study limitations is the fact that it uses a specific moment of a specific year and that hospital infection and antimicrobial resistance rates are prevalence and not incidence rates. However, resistance data obtained from the Point Prevalence Study largely reflects incidence data obtained in the EARS-Net [17].

The same temporal scale was considered for all the countries and variables in this study (the year 2016 with impact in HAI and Percentual AMR in 2016 and 2017). Regarding spatial scale, and considering the European region studied as one spatial region, the aggregation bias may be considered diluted as data collection included 23 data collection location points (countries).

Regarding socio-economic data of 2016, it does not significantly deviate from data collected for previous years in each country.

We were not able to find a relation between veterinary antimicrobial consumption and AMR rate. According to ESVAC report for 2016 [18], variations in both the sales patterns and magnitudes of sales of these products may be due to differences, between the countries, in the relative proportion of the various food-producing animal species, the availability of veterinary antimicrobial products, and the general situation concerning infectious diseases. However, the report's authors agree that these factors cannot fully explain the differences.

No database was found for agriculture consumption of antibiotics or for resistant bacteria in animals and vegetables at the European Union level. However, a recent publication shows the presence of the plasmid-mediated colistin resistance (PMCR)—encoding gene mcr-1—in an Escherichia coli isolate, recovered from lettuce produced and marketed in Portugal [19]. Colistin is a last-resource antibiotic used for infections caused by multidrug-resistant bacteria. Lettuce is a vegetable commonly consumed fresh and not subjected to any cooking process, which may amplify human food safety risks in terms of AMR transmission. The detection of a mobile colistin resistance gene in a raw vegetable may constitute a serious public health concern and reinforce the need for a One Health surveillance and action approach.

## 5. Conclusions

Our study shows that higher human ambulatory consumption of antibiotics, lower per capita expenditure on health, and a higher percentage of private health expenditure are associated with a higher rate of antimicrobial resistance. Low per capita expenditure on health may be a surrogate of worse healthcare conditions—infrastructure, human resources, and organization. A high percentage of private health expenditure may be a surrogate of less regulated antimicrobial prescriptions.

Our findings may have major policy implications. It may be that a quasi-total focus put on reducing human and animal antibiotic consumption as the most important factors contributing to antimicrobial resistance may be a deviant focus, as resistance transmission may be paramount for antimicrobial resistance levels. Therefore, increasing public expenditure on healthcare, promoting interventions to improve sanitation, infection control and prevention, adequate human resources, better infrastructure, and One Health governance are jointly necessary to tackle antimicrobial resistance at the European scale. 

## Figures and Tables

**Table 1:** Descriptive statistics of the variables included in the study.

	Minimum	Maximum	Mean	SD	n
Prevalence of healthcare associated infections (HAI), %	2.95	8.10	4.96	1.55	23
Bacteria resistant to antibiotic (Resistant), %	10.69	68.42	29.9251	15.72056	21
Human antibiotic consumption at ambulatory care, DID	9.20	31.00	17.4591	5.74669	22
Human antibiotic consumption at hospital care, DID	0.85	2.38	1.7226	0.43679	19
Human antibiotic consumption (total), DID	10	33	19.70	6.094	19
Veterinary antibiotic overall sales, Tonnes	0.59	2726.54	315.95	608.08	23
Veterinary antibiotic sales for food-producing animals, Tonnes	0.56	2724.89	314.55	620.95	22
Veterinary antibiotic sales population correction unit, 1000 Tonnes	54.62	8734.01	2156.80	2630.95	22
Total expenditure on health, per capita,	715.95	5785.41	2700.79	1605.21	23
Total expenditure on health, % of GDP	5.47	11.48	8.48	1.73	23
Government expenditure on health, % of GDP	3.40	9.54	6.40	1.70	23
Years lost, /100,000 population, aged 75 years old	3110.00	9122.00	4629.18	1668.09	17

**Table 2:** Percentual AMR Correlation with the remaining variables included in the study.

	% Resistant		n
	Linear Correlation	Coefficients	
Prevalence of healthcare associated infections (HAI), %	r = 0.154 (*p* = 0.506)	rs = 0.045 (*p* = 0.845)	21
Human antibiotic consumption at ambulatory care, DID	r = 0.339 (*p* = 0.144)	rs = 0.387 (*p* = 0.092)	20
Human antibiotic consumption at hospital care, DID	r = 0.171 (*p* = 0.497)	rs = 0.164 (*p* = 0.515)	18
Human antibiotic consumption (total), DID	r = 0.275 (*p* = 0.269)	rs = 0.305 (*p* = 0.219)	18
Veterinary antibiotic overall sales, Tonnes	r = 0.037 (*p* = 0.873)	rs = 0.187 (*p* = 0.417)	21
Veterinary antibiotic sales for food-producing animals, Tonnes	r = 0.036 (*p* = 0.879)	rs = 0.188 (*p* = 0.427)	20
Veterinary antibiotic sales population correction unit, 1000 Tonnes	r = -0.124 (*p* = 0.603)	rs = 0.104 (*p* = 0.663)	20
Total expenditure on health, per capita, €	r = -0.675 ** (*p* = 0.001)	rs = −0.722 ** (*p* < 0.001)	21
Total expenditure on health, % of GDP	r = −0.428 (*p* = 0.053)	rs = −0.435 * (*p* = 0.049)	21
Government expenditure on health, % of GDP	r = −0.597 ** (*p* = 0.004)	rs = −0.534 * (*p* = 0.013)	21
Years lost /100,000 population, aged 75 years old	r = 0.305 (*p* = 0.269)	rs = 0.564 * (*p* = 0.028)	15

**—Correlation is significant at the 0.05 level (2-tailed). *—Correlation is significant at the 0.05 level (2-tailed). r—Pearson’s R; rs—Spearman’s Rho.

**Table 3:** Multiple linear regression model for % AMR rate.

Variables	Unstandardized Coefficients		Standardized Coefficients	t	*p*
	B	Std. Error	Beta		
(Constant)	19.741	8.275		2.386	0.044
Total expenditure on health, per capita, €	−0.007	0.002	−0.668	−4.158	0.003
Human antibiotic consumption at ambulatory care, DID	1.412	0.383	0.591	3.683	0.006
Dependent Variable: % AMR rate R^2^ = 0.891; R^2^ adj = 0.742					

## Data Availability

Data is contained within the article or supplementary material.

## Supplementary Materials

The following are available online at https://www.mdpi.com/article/10.3390/antibiotics10070834/s1, Figure S1: Scatterplot, linear and cubic regression models (95% confidence intervals) for the relation between percentual AMR rate with ambulatory antimicrobial consumption, Figure S2: Scatterplot, linear and cubic regression models (95% confidence intervals) for the relation between percentual AMR rate with years of life lost rate per 100,000 inhabitants aged 75 years old or more, Table S1: Correlation of rate of AMR with the remaining variables included in the study after adjustment for Expenditure on health, per capita, €, Table S2: Multiple linear regression model for AMR rate adjusting for polynomial relation with expenditure on health per capita.
