# Supplementary material for: Determinants of Antimicrobial Resistance among the Different European Countries: More than Human and Animal Antimicrobial Consumption
Source: Antibiotics (Basel). 2021 Jul 9;10(7):834. doi: 10.3390/antibiotics10070834 (PMC8300618; doi:10.3390/antibiotics10070834)
Supplement: Supplementary file 1 [file antibiotics-10-00834-s001.zip › antibiotics-1254449-SI.pdf]

Supplementary Materials

Table S1. Correlation of rate of AMR with the remaining variables included in the study after adjustment for Expenditure on health, per capita, €.

	% Resistant	
	**Partial correlation**	**df**
**Prevalence of healthcare associated infections (HAI),** %	r = 0.524 (*p* = 0.120)	8
**Human antibiotic consumption at ambulatory care,** DID	r = 0.793 (*p* = 0.006**)	8
**Human antibiotic consumption at hospital care,** DID	r = 0.100 (*p* = 0.783)	8
**Human antibiotic consumption (total),** DID	r = 0.776 (*p* = 0.008**)	8
**Veterinary antibiotic overall sales,** Tonnes	r = 0.025 (*p* = 0.946)	8
**Veterinary antibiotic sales for food-producing animals,** Tonnes	r = 0.025 (*p* = 0.946)	8
**Veterinary antibiotic sales population correction unit,** 1000 Tonnes	r = 0.128 (*p* = 0.725)	8
**Total expenditure on health,** % of GDP	r = 0.076 (*p* = 0.835)	8
**Government expenditure on health,** % of GDP	r = -0.152 (*p* = 0.675)	8
**Years lost, /100,000 population, aged 75 years old**	r = -0.467 (*p* = 0.174)	8

Table S2. Multiple linear regression model for AMR rate adjusting for polynomial relation with expenditure on health per capita.

Block	**Variables**	**Unstandardized Coefficients**		**Standardized Coefficients**	**t**	***p***
		**B**	**Std. Error**	**Beta**		
Block 1 (enter method)	**(Constant)**	7.454	9.350		0.797	0.461
	**Expenditure on health, per capita, €**	−0.037	0.014	−3.649	−2.569	0.050
	**Squared Expenditure on health, per capita, €**	4.837×10^{-6}	0.000	2.639	0.887	0.416
	**Cubic Expenditure on health, per capita, €**	2.825×10^{-6}	0.000	0.746	0.443	0.676
Block 2 (stepwise)	**Private Expenditure on health, %GDP**	19.694	3.782	0.798	5.207	0.003
	**Human antibiotic consumption at ambulatory care,** DID	0.984	0.232	0.412	4.249	0.008

Private Expenditure on health, %GDP = Expenditure on health, %GDP—Gov expenditure on health, %GDP

Dependent Variable: AMR rate;

$R^2 = 0.986$; R^2 adj = 0.943

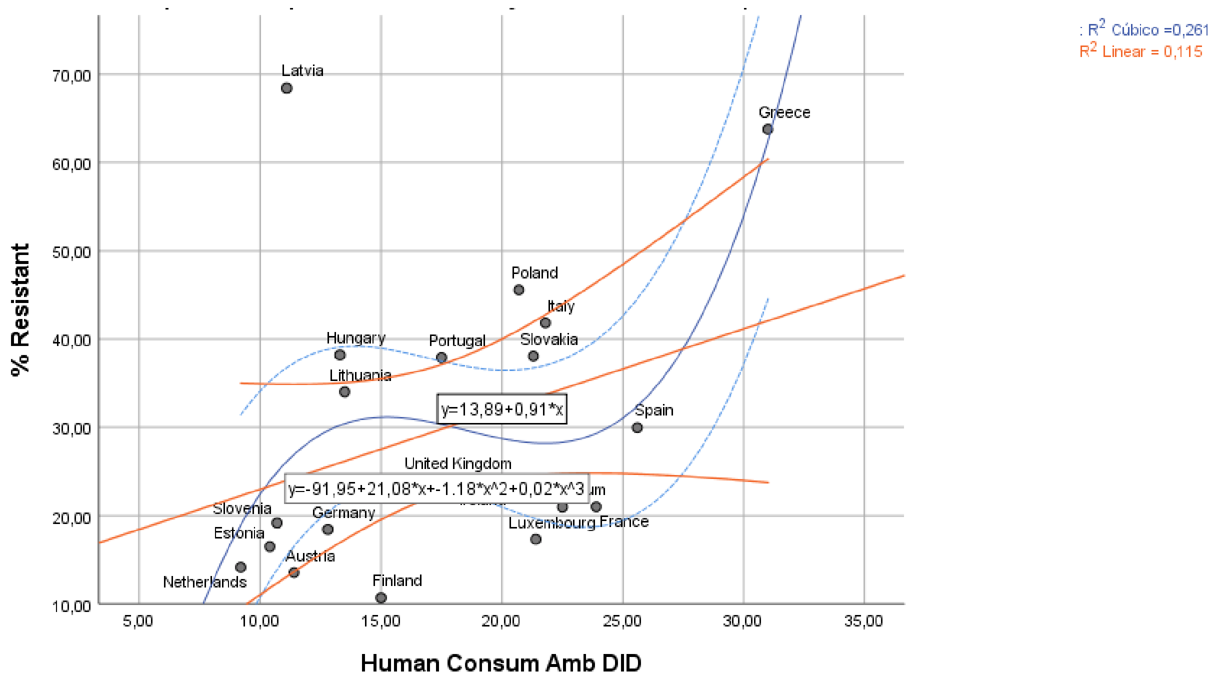

Figure S1. Scatterplot, linear and cubic regression models (95% confidence intervals) for the relation between percentual AMR rate with ambulatory antimicrobial consumption.

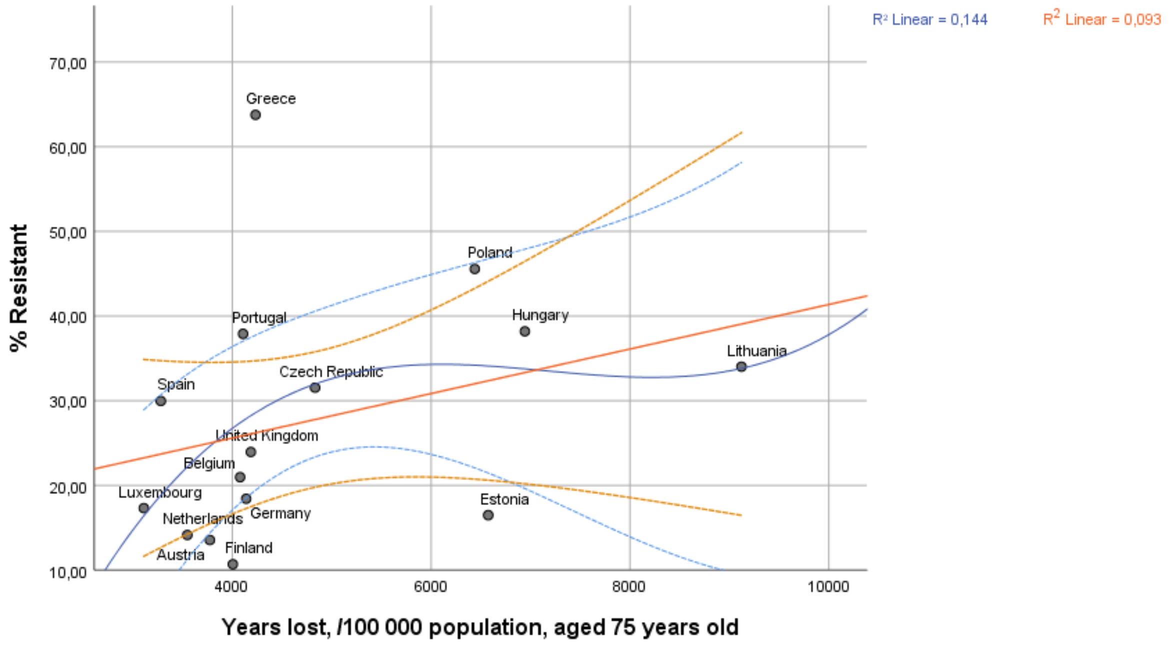

Figure S2. Scatterplot, linear and cubic regression models (95% confidence intervals) for the relation between percentual AMR rate with years of life lost rate per 100,000 inhabitants aged 75 years old or more.
